# The association between Vapor Pressure Deficit and arthritis: Evidence from a 10-year longitudinal study of middle-aged and elderly Chinese adults

**DOI:** 10.1371/journal.pone.0344015

**Published:** 2026-03-20

**Authors:** Damei Ye, Shuchao Ye, Changyi Lin, Dongming Lu, Xuelan You, Chaoyan Xu, Yongyang Wu

**Affiliations:** 1 Department of Rheumatology Immunology, Affiliated Sanming First Hospital, Fujian Medical University, Sanming, Fujian, China; 2 Department of Urology, Affiliated Sanming First Hospital, Fujian Medical University, Sanming, Fujian, China; Regional Health Care and Social Agency of Lodi, ITALY

## Abstract

**Objective:**

To investigate the association between vapor pressure deficit (VPD) and arthritis in middle-aged and older Chinese adults.

**Methods:**

This study utilized data from the China Health and Retirement Longitudinal Survey (CHARLS) spanning the years 2011–2020. Participants without arthritis in 2011 were selected as the study population, with VPD designated as the primary exposure factor and newly diagnosed arthritis cases as the outcome variable. Logistic regression models were used to estimate the association between VPD and incident arthritis. Restricted cubic spline (RCS) analyses were conducted to assess potential nonlinearity. Subgroup analyses were performed to examine effect heterogeneity across population subgroups.

**Results:**

A total of 4615 subjects were included, and a total of 1317 subjects were reported to be diagnosed with arthritis during approximately 10 years of follow-up (2011–2020). The VPD level in the arthritis group was lower than that in the non-arthritis group (5.184 ± 0.828 vs 5.291 ± 0.818, p < 0.001). All logistic regression models showed that VPD was linearly related to the incidence of arthritis, and the relationship remained consistent even when VPD was categorized. RCS analysis showed that the incidence of arthritis decreased significantly with increasing VPD (p < 0.05), especially when VPD was lower than 5.28. Subgroup analysis indicated that VPD exerted a stronger protective effect against arthritis among rural residents (p for interaction = 0.006).

**Conclusion:**

VPD was found to be negatively associated with the incidence of arthritis among middle-aged and elderly populations, with a particularly stronger effect observed in rural residents. These findings highlight VPD as an environmental factor associated with arthritis and may help improve understanding of environmental influences on arthritis development.

## Introduction

Arthritis, a common musculoskeletal disorder, presents a significant public health challenge, imposing substantial economic and medical burdens [1–3]. Osteoarthritis (OA) and rheumatoid arthritis (RA) are among the most prevalent forms. Globally, arthritis affects an estimated 355 million people, with 190 million having OA and over 16.5 million suffering from RA [4,5]. These conditions cause joint and synovial tissue inflammation, leading to symptoms like swelling, pain, and stiffness, and may result in joint deformities [6,7]. In China, the number of arthritis patients exceeds 100 million and is rising [2,8].

Environmental factors, particularly weather conditions, have long been considered significant in influencing joint pain. As early as 1986, research indicated that the severity of joint pain was related to weather conditions [9]. Subsequent studies further confirmed the correlation between weather temperature, relative humidity, atmospheric pressure, and precipitation with arthritis [10–13]. However, some studies found no association between these weather conditions and the risk of joint pain [14]. Despite extensive research, the findings remain largely contradictory. Our longitudinal design with objective VPD measurements from high-resolution atmospheric data addresses key limitations of previous studies, including reliance on subjective weather perceptions and cross-sectional designs that cannot establish temporal relationships. Therefore, it is crucial to comprehensively study the relationship between weather conditions and arthritis, as this has profound implications for the prevention and management of arthritis.

Humidity is one of the most frequently studied climatic factors. An animal experiment showed that high humidity conditions (80 ± 5%) can exacerbate arthritis [15]. A small-scale human study suggests that climatic factors such as temperature and atmospheric pressure may influence T/B cell subset ratios and potentially trigger autoimmunity in RA (rheumatoid arthritis) [16]. Additionally, research has found a weak positive correlation between osteoarthritis (OA) pain and humidity (the higher the humidity, the greater the pain) [13]. Therefore, humidity is closely related to the occurrence of arthritis.

A first 1 km high-resolution atmospheric moisture index collection over China (HiMIC-Monthly) includes six common atmospheric moisture indices: Relative Humidity (RH), Actual Vapor Pressure (AVP), Vapor Pressure Deficit (VPD), Dew Point Temperature (DPT), Mixing Ratio (MR), and Specific Humidity (SH) [17]. Vapor Pressure Deficit (VPD) is the difference between the actual vapor pressure and the saturated vapor pressure of air, which quantifies the degree of the air’s undersaturation with water vapor. A larger VPD value indicates drier air, while a smaller value indicates moister air. The total R2 values for these indices in HiMIC-Monthly exceed 0.96, and both the root mean square error and mean absolute error are within reasonable ranges. The dataset uses the Albers Equal Area Conic projection, with a spatial resolution of 1 km × 1 km and a temporal resolution of one month, covering mainland China from January 2003 to December 2020. Although these indices have been extensively studied in meteorology, their association with arthritis remains largely unexplored.

The China Health and Retirement Longitudinal Study (CHARLS) is a large-scale, nationwide longitudinal project aimed at assessing the health status and retirement issues of the elderly in China. The CHARLS database provides comprehensive individual information, including demographics, health conditions, and lifestyles. To our knowledge, this is the first study to link the HiMIC‑Monthly high‑resolution atmospheric humidity dataset with the nationwide CHARLS cohort of older adults to examine the relationship between environmental humidity and new‑onset arthritis. The primary aim was to evaluate the association between VPD and risk of new‑onset arthritis.

This study utilizes the CHARLS population and HiMIC-Monthly data to explore the potential link between atmospheric moisture indices and arthritis, aiming to provide new perspectives and strategies for the prevention and treatment of arthritis.

## Materials and methods

### 1. Study design

CHARLS, implemented by the National School of Development at Peking University, is a long-term cohort study. The detailed project description and the questionnaire have been published elsewhere [18]. By employing a multi-stage sampling strategy, the study aims to minimize sample selection bias and enhance representativeness. Focusing on residents aged 45 and above in mainland China, CHARLS aims to build a comprehensive public database containing rich socioeconomic and health data. The nationwide baseline survey commenced in 2011, followed by tracking surveys every two to three years. The study covers 150 county-level administrative units, 450 villages, and approximately 17,000 individuals from 10,000 households.

### 2. Participants

**Fig 1:**
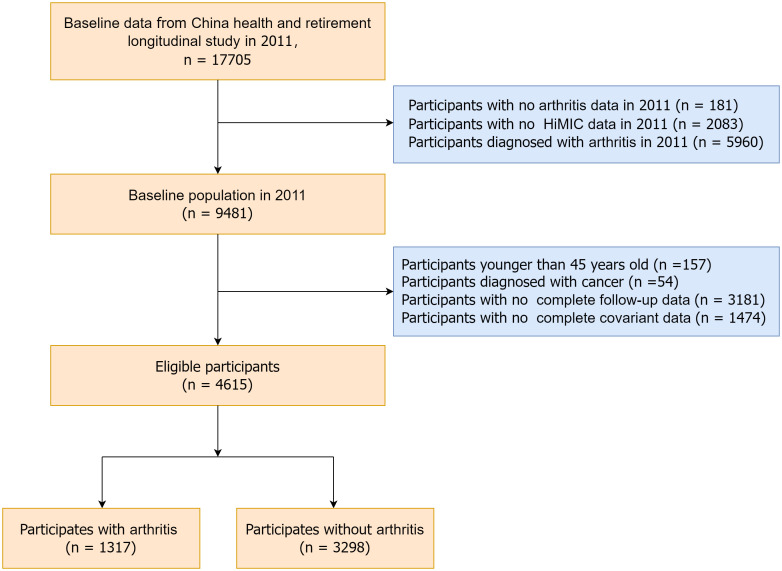
Flow chart of participant selection from the China Health and Retirement Longitudinal Study (CHARLS).

We utilized baseline data from CHARLS participants in 2011 (n = 17,705). A total of 181 participants with missing arthritis questionnaire data, 5,960 participants with a prior diagnosis of arthritis, and 2,083 participants with missing atmospheric humidity index data were excluded. Follow-up assessments were conducted in 2013, 2015, 2018, and 2020. Further exclusions included 157 participants younger than 45 years, 54 participants with a history of cancer, 3,181 participants without endpoint events or complete follow-up data, and 1,474 participants with incomplete covariate data. Ultimately, 4,615 participants were included in the final analysis. A detailed recruitment flow chart is presented in Fig 1.

### 3. Variables

The outcome was incident arthritis during follow-up. Among participants without arthritis at baseline (2011), incident cases were identified in the 2013, 2015, 2018, and 2020 waves using the CHARLS binary question “Did the doctor tell you that you have arthritis?” A “Yes” response classified the participant as having physician-diagnosed arthritis and a “No” response as non-arthritis. This self-reported physician diagnosis is a common approach in large-scale epidemiological studies and, although it may introduce misclassification bias, its focus on doctor diagnosis rather than self-reported symptoms likely improves reliability. CHARLS does not specify joint site or distinguish arthritis subtypes (e.g., osteoarthritis vs. rheumatoid arthritis), so we use the broader term “arthritis” throughout the manuscript; the inability to differentiate types is a key limitation because etiologies and responses to environmental factors may vary by subtype.

The main exposure variables are the atmospheric humidity indices in the subjects’ living environments, including RH, AVP, VPD, DPT, MR, and SH. These indices are based on the average values from January to December 2011, as provided by HiMIC-Monthly. VPD values were extracted based on participants’ residential addresses at the county level using the 1 km × 1 km resolution HiMIC-Monthly dataset. To minimize seasonal variation bias, we calculated the annual average VPD from January to December 2011. VPD values were matched to participants based on county-level residential codes in CHARLS, which were linked to the corresponding 1 km × 1 km grid cells in HiMIC-Monthly using spatial overlay techniques. The HiMIC data are publicly available on the internet for data users and researchers throughout the world [17].

Demographic and socioeconomic characteristics included age, sex, ethnicity, marital status, education level, and living environment (categorized as rural or urban). Lifestyle factors assessed were smoking status (defined as having smoked more than 100 cigarettes in a lifetime), alcohol, body mass index (BMI), and nightly sleep duration. Additionally, the study accounted for participants’ comorbidities, including hypertension, diabetes, and cardiovascular disease.

### 4. Statistical analysis

All statistical analyses were performed using R version 4.4.1 (R Foundation for Statistical Computing, Vienna, Austria), with statistical significance rigorously established at P < 0.05. Continuous variables within the baseline table are delineated by their mean values (standard error), whereas categorical variables are depicted by their respective sample sizes (percentages).

Logistic regression models were used to estimate the association between atmospheric humidity indices and incident arthritis. Restricted cubic spline (RCS) regression was applied to assess potential nonlinearity. Sequentially adjusted models were fitted to evaluate the robustness of the VPD–arthritis association after controlling for potential confounders. All analyses focused on effect estimation for the VPD–arthritis association rather than individual-level risk prediction.

### 5. Ethics statement

This study was performed in line with the principles of the Declaration of Helsinki. Peking University Biomedical Ethics Review Committee (approval number: IRB00001052–11015) granted CHARLS the ethical approval. Before joining the group, each participant provided a written statement of informed consent. Because the data is public, this study does not need further ethical approval. CHARLS project website provides downloadable data and information (http://charls.pku.edu.cn).

## Results

### 1. Characteristics of the study population

A total of 4,615 subjects without arthritis in 2011 were included in the study, among whom 1,317 were diagnosed with arthritis during approximately 10 years of follow-up (2011–2020). The analysis revealed that the atmospheric humidity index, VPD, was significantly associated with the incidence of arthritis. The VPD level in the living environment of the arthritis group was lower than that of the non-arthritis group (5.184 ± 0.828 vs. 5.291 ± 0.818, p < 0.001). However, the other humidity indices (RH, AVP, DPT, MR, and SH) did not differ significantly between participants with and without incident arthritis (p = 0.244, 0.611, 0.053, 0.893, and 0.898, respectively). It is noteworthy that participants in the arthritis group were older (58.647 ± 9.021 vs. 57.393 ± 8.717, p < 0.001) and had shorter nighttime sleep duration (6.365 ± 1.839 vs. 6.645 ± 1.709, p < 0.001). The arthritis group also had lower proportions of males, individuals of Han ethnicity, those who were married, those with a high school education or above, urban resident, smokers, drinkers, and participants without cardiovascular disease. However, there were no significant differences between the groups in terms of body mass index (BMI), hypertension, or diabetes (p > 0.05). The baseline characteristics of the study population are summarized in Table 1.

**Table 1:** Characteristics of participants by arthritis.

Variable	Total (n = 4615)	Arthritis (n = 1317)	non-Arthritis (n = 3298)	P value
**Age**	57.751 ± 8.822	58.647 ± 9.021	57.393 ± 8.717	<0.001
**Age group**				<0.01
< 60 years old	2826 (61.235)	759 (57.631)	2067 (62.674)	
≥ 60 years old	1789 (38.765)	558 (42.369)	1231 (37.326)	
**Sex**				<0.001
male	2273 (49.252)	589 (44.723)	1684 (51.061)	
female	2342 (50.748)	728 (55.277)	1614 (48.939)	
**Race**				<0.001
han	4360 (94.475)	1212 (92.027)	3148 (95.452)	
others	255 (5.525)	105 (7.973)	150 (4.548)	
**Marital Status**				<0.01
Non-Married	449 (9.729)	158 (11.997)	291 (8.824)	
Married	4166 (90.271)	1159 (88.003)	3007 (91.176)	
**Educational Level**				<0.001
Elementary school and below	2860 (61.972)	904 (68.641)	1956 (59.309)	
High school and higher	1755 (38.028)	413 (31.359)	1342 (40.691)	
**Residence Place**				<0.01
rural	2913 (63.120)	880 (66.819)	2033 (61.643)	
urban	1702 (36.880)	437 (33.181)	1265 (38.357)	
**Smoke**				0.049
no	2761 (59.827)	818 (62.111)	1943 (58.914)	
yes	1854 (40.173)	499 (37.889)	1355 (41.086)	
**Alcohol**				<0.001
no	3018 (65.395)	914 (69.400)	2104 (63.796)	
yes	1597 (34.605)	403 (30.600)	1194 (36.204)	
**Body mass index**	23.707 ± 3.847	23.790 ± 3.830	23.674 ± 3.853	0.352
**Sleep duration at night (hours)**	6.565 ± 1.752	6.365 ± 1.839	6.645 ± 1.709	<0.001
**Hypertension**				0.123
no	3606 (78.137)	1009 (76.614)	2597 (78.745)	
yes	1009 (21.863)	308 (23.386)	701 (21.255)	
**Diabetes mellitus**				0.735
no	4381 (94.930)	1253 (95.140)	3128 (94.845)	
yes	234 (5.070)	64 (4.860)	170 (5.155)	
**Cardiovascular disease**				0.013
no	4180 (90.574)	1170 (88.838)	3010 (91.267)	
yes	435 (9.426)	147 (11.162)	288 (8.733)	
**Relative humidity**	69.845 ± 8.057	70.069 ± 8.391	69.755 ± 7.920	0.244
**Actual Vapor Pressure**	13.898 ± 4.254	13.846 ± 4.418	13.919 ± 4.188	0.611
**Vapor Pressure Deficit**	5.260 ± 0.822	5.184 ± 0.828	5.291 ± 0.818	<0.001
**Dew Point Temperature**	12.282 ± 2.692	12.157 ± 2.844	12.332 ± 2.627	0.053
**Mixing Ratio**	9.032 ± 2.644	9.041 ± 2.739	9.029 ± 2.606	0.893
**Specific Humidity**	8.916 ± 2.592	8.924 ± 2.685	8.913 ± 2.554	0.898

### 2. Relationship between VPD and arthritis

Three logistic regression models were constructed to examine the relationship between VPD and arthritis incidence. Model 1 was unadjusted, with no covariates included. Model 2 was adjusted for demographic and socioeconomic characteristics with p-values < 0.05, including Age, Sex, Race, MaritalStatus, Educational Level and Residence Place. Model 3 was further adjusted to include additional factors with p-values < 0.05, encompassing Age, Sex, Race, MaritalStatus, Educational Level, Residence Place, Smoke, Alcohol, Cardiovascular disease and Sleep duration at night. A linear relationship between VPD and arthritis incidence was observed across all three models (OR = 0.85 [0.79, 0.92]; OR = 0.88 [0.81, 0.95]; OR = 0.89 [0.82, 0.96]). This association remained significant even after categorizing VPD (p < 0.05). Specifically, arthritis incidence decreased by 31%, 25%, and 24% in high VPD environments in Models 1, 2, and 3, respectively. Table 2 illustrates the relationship between VPD and arthritis incidence.

**Table 2:** Association of vapor pressure deficit with arthritis.

Exposure	Model 1		Model 2		Model 3	
	95%CI	P value	95%CI	P value	95%CI	P value
Vapor Pressure Deficit	0.85 (0.79,0.92)	<0.001	0.88 (0.81,0.95)	0.002	0.89 (0.82,0.96)	0.003
Vapor Pressure Deficit						
Q1 [3.33,4.66]	reference		reference		reference	
Q2 (4.66,5.28]	0.77 (0.65,0.92)	0.004	0.83 (0.69,1.00)	0.050	0.84 (0.70,1.01)	0.060
Q3 (5.28,5.95]	0.82 (0.69,0.99)	0.040	0.87 (0.72,1.05)	0.130	0.87 (0.72,1.05)	0.140
Q4 (5.95,6.98]	0.69 (0.58,0.82)	<0.001	0.75 (0.63,0.90)	0.002	0.76 (0.64,0.91)	0.003
P for trend		<0.001		0.010		0.010

Model 1 was adjusted for no covariates.

Model 2 was adjusted for Age, Sex, Race, Marital Status, Educational Level, Residence Place.

Model 3 was adjusted for Model 2 plus Smoke, Alcohol, Cardiovascular disease, Sleep duration at night.

**Fig 2:**
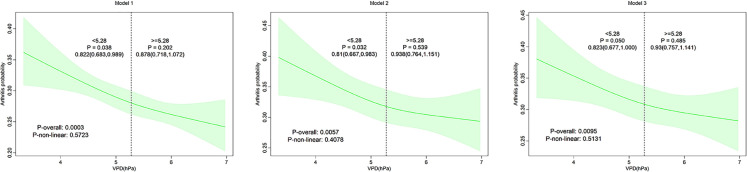
Restricted cubic spline (RCS) analysis showing the relationship between Vapor Pressure Deficit (VPD) and arthritis incidence in three models.

RCS analysis (Fig 2) showed that there was no nonlinear relationship between VPD and arthritis incidence in the three models (P-nonlinearity > 0.05). However, with the increase of VPD, the incidence of arthritis decreased significantly (p = 0.0003, p = 0.0057, and p = 0.0095, respectively). While the overall relationship was linear (p-nonlinearity > 0.05), the protective effect appeared more pronounced at VPD values below 5.28, suggesting a potential inflection point worthy of future investigation, with OR values of 0.822 [0.683–0.989], 0.810 [0.667–0.983], and 0.823 [0.677–1.000], respectively.

**Fig 3:**
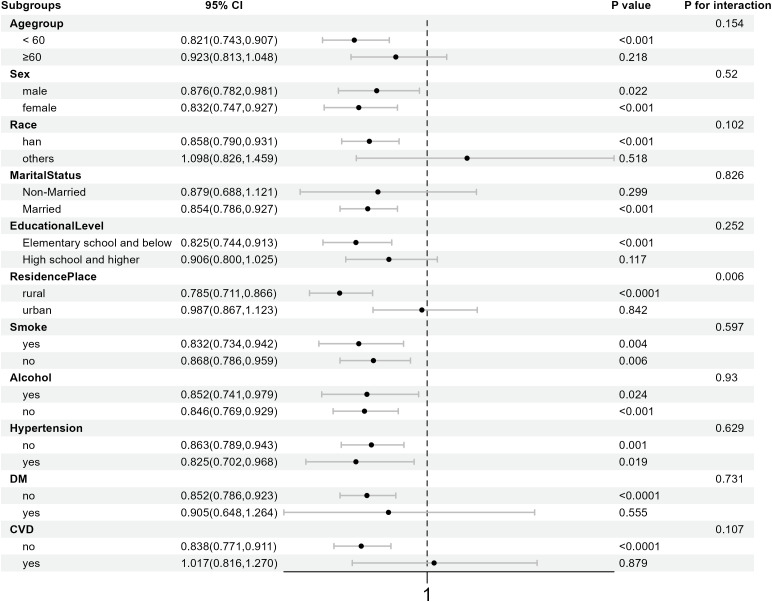
Forest plot of subgroup analysis examining the association between VPD and arthritis incidence across different populations.

Subgroup analysis was conducted to evaluate the consistency of the association between VPD and arthritis incidence across different populations (Fig 3). Multivariate logistic regression analysis was performed within different groups after adjusting for age, sex, race, marital status, education level, place of residence, smoking, drinking, hypertension, diabetes, and CVD. Subgroup analysis showed that for rural residents, the protective effect of increased VPD in the living environment on arthritis was more obvious (p = 0.006). The stronger protective effect in rural residents should be interpreted cautiously as rural/urban residence may proxy for unmeasured factors including occupational exposures, air quality, lifestyle factors, and healthcare access.

## Discussion

Drawing upon a comprehensive national cohort study encompassing 17,000 samples across various provinces in China, our research demonstrates that an increase in the VPD of the atmospheric humidity index is associated with a decrease in the incidence of new arthritis cases, even after accounting for potential confounding factors. In this nationwide longitudinal cohort, higher VPD (drier air) was associated with a lower incidence of new-onset arthritis after adjustment for demographic, socioeconomic, lifestyle, and comorbidity covariates. These findings suggest that ambient air dryness, as captured by VPD, may be an environmental factor relevant to arthritis development.

The elevation in air pollutant concentrations has been linked to an increased risk of mortality from all causes, including cardiovascular, respiratory, vascular, and cancer-related deaths [19]. Air pollution significantly contributes to the development of seropositive rheumatoid arthritis, and the rise in air pollution and climate change may drive up the incidence of rheumatoid arthritis [20]. Additionally, an increase in environmental temperature has been associated with a higher incidence of arrhythmia, cardiac arrest, and coronary heart disease [21]. Consequently, weather parameters are intricately linked to chronic health conditions and heightened risks of adverse health events. Osteoarthritis (OA) and rheumatoid arthritis (RA) are the most prevalent musculoskeletal disorders, and it is widely believed that weather factors can trigger new onset or exacerbate symptoms of these condition [22,23]. Notably, over half of OA patients report that their pain is weather-induced [11,24]. Despite this widespread belief, past research has yielded contradictory findings. Studies by Manuela L. Ferreira and others indicate that air temperature, humidity, pressure, and rainfall do not significantly heighten the risk of new or sudden musculoskeletal pain onset [14]. However, many of these studies suffer from methodological limitations, such as reliance on self-reported data and perceptions of weather changes, and insufficient adjustment for potential confounders. To address these limitations, we selected non-arthritic individuals over 45 years old from the CHARLS in 2011, recorded their atmospheric humidity index in the living environment, and followed them for a decade to ascertain the incidence of new arthritis cases. This approach helps overcome previous constraints and clarifies the causal relationship. The results demonstrated that VPD, an indicator of atmospheric humidity, was negatively correlated with new-onset arthritis.

VPD is an index that measures the difference between the actual water vapor content in the air and the saturated water vapor content, thus reflecting the degree of air dryness. Fluctuations in atmospheric moisture content have significant implications for human living environments and public health [25]. For example, during periods of high temperatures, elevated humidity can hinder the body’s ability to dissipate heat, thereby increasing the risk of heatstroke and related health conditions [26–28]. Additionally, changes in humidity levels can facilitate the transmission of diseases such as influenza, malaria, and dengue fever [29,30]. Notably, our research uncovers a novel relationship between atmospheric moisture and certain diseases, revealing that an increase in VPD is associated with a decrease in the incidence of new arthritis cases. Our study indicates that VPD is inversely associated with incident arthritis among middle-aged and older adults, particularly among rural residents.

Recent evidence suggests that environmental factors play crucial roles in inflammatory conditions [31], supporting the plausibility of our findings. The body’s nutritional status is a central regulator of inflammatory responses, and specific nutrients and dietary components can substantially influence health by modulating molecular pathways such as oxidative stress, cellular signaling, and the synthesis of inflammatory mediators [32]. Accordingly, the protective effect of VPD is likely co-mediated through nutrition-related anti-inflammatory pathways. Notably, we observed that the protective association between VPD and arthritis was more pronounced among rural residents. Beyond differences in physical activity and exposure to environmental pollutants, variation in dietary patterns may be an important effect modifier. For example, among non-hypertensive adults, greater adherence to the Dietary Approaches to Stop Hypertension (DASH) and the Mediterranean diet is associated with lower systolic blood pressure and a reduced likelihood of prehypertension [33]. Moreover, in a cross-sectional study of nearly 10,000 Iranian adults, higher intakes of total polyphenols, phenolic acids, and lignans were linked to lower odds of non-alcoholic fatty liver disease (NAFLD), suggesting that diets rich in these polyphenols may reduce NAFLD risk [34]. Taken together, rural residents’ predominantly plant-based diets may act synergistically with the environmental effects of VPD to confer stronger protective benefits.

Our findings have potential implications for climate adaptation strategies in China. As climate change alters regional humidity patterns, understanding VPD-health relationships could inform public health planning, particularly for aging populations in regions experiencing increasing aridity.

This study found that higher VPD (drier air) was associated with a lower incidence of arthritis, which on the surface differs from prior studies that focus on symptom fluctuations. Existing literature [35–38] provides indirect biological plausibility for this association: humidity changes can affect skin and mucosal barrier function and modulate local/systemic immune and inflammatory responses; VPD is also related to temperature, sunlight (which affects vitamin D synthesis), and outdoor activity levels, all of which may act as confounders or mediators. However, these studies have not directly tested causal pathways from VPD to arthritis onset and therefore cannot be taken as direct evidence. Our study is based on an existing database and lacks individual-level data on sunlight exposure, outdoor activity, and inflammatory biomarkers, so we cannot verify specific mechanisms within this analysis. Future prospective cohort or mechanistic studies that include individual exposure assessment and biomarker measurements are needed to further confirm and elucidate these potential pathways.

Our research presents several advantages. Firstly, the CHARLS encompasses a wide array of regions across China and boasts a substantial sample size, enhancing the representativeness and credibility of the findings. Secondly, CHARLS employs a longitudinal research design, allowing for the tracking of the same cohort’s health status over time and facilitating a more precise evaluation of the relationship between various VPD indicators and the incidence of arthritis. Thirdly, stringent quality control measures were implemented during data collection to ensure accuracy and reliability. Lastly, the study not only examines the link between VPD and arthritis incidence but also accounts for potential confounding factors, including demographic and socio-economic characteristics, lifestyle factors and chronic diseases, rendering the analysis more comprehensive. Finally, we assessed the robustness of the VPD–arthritis association using sequentially adjusted logistic regression models, RCS analyses, and subgroup analyses

Nevertheless, our study has several limitations. First, VPD measurement may be influenced by regional and seasonal variation; although we used annual averages and the high-resolution (1 km x 1 km) HiMIC-Monthly dataset to reduce these effects, residual confounding from microenvironmental factors cannot be completely ruled out. Second, arthritis diagnosis relied entirely on self-reported physician diagnosis without clinical validation (no DAS28, X-ray/MRI, or ultrasound), which may introduce non-differential misclassification and attenuate the true effect. Third, the heterogeneity of arthritis types is a major limitation: osteoarthritis and rheumatoid arthritis have distinct pathophysiologies and may respond differently to VPD, but CHARLS does not allow subtype differentiation. We adjusted for BMI, education, urban/rural residence, marital status, and other covariates to partially control confounding, but detailed occupational exposures (e.g., long-term heavy physical labor, joint-specific loading) and healthcare-access indicators (e.g., access to rheumatology specialists) were unavailable, so residual confounding cannot be excluded. Future prospective studies should combine hospital records, imaging/ultrasound scoring, disease-activity measures (e.g., DAS28), inflammatory biomarkers, and more detailed occupational and healthcare-resource data to objectively validate and subtype incident arthritis and to confirm and refine the observed VPD-arthritis association.

In conclusion, our study demonstrates that VPD is negatively associated with incident arthritis among middle-aged and older adults, with a stronger association observed in rural residents. This finding provides new insights into the environmental epidemiology of arthritis, and further large-scale and mechanistic studies are needed to confirm these findings and clarify potential biological pathways.
